# C-751-02. A Systematic Review of Using Digital Phenotyping in Trauma Populations

**DOI:** 10.1093/jbcr/irag033.007

**Published:** 2026-04-06

**Authors:** Sheehan Choudhury, Huan Deng, Mary Butler, Sarah C Hutton, Daniel S Barron, Colleen M Ryan, Juan P Herrera-Escobar, Stephanie F Ford, Jeffrey C Schneider

**Affiliations:** Spaulding Rehabilitation Hospital, Harvard Medical School, Boston, MA; Spaulding Rehabilitation Hospital, Harvard Medical School, Boston, MA; Graduate from UMass Amherst, Brighton, MA; University of Massachusetts T.H Chan School of Medicine, Boston, MA; Brigham and Women's Hospital, Harvard Medical School, Boston, MA; Massachusetts General Hospital, Harvard Medical School, Boston, MA; Department of Surgery, Brigham and Women's Hospital, Harvard Medical School, Boston, MA; Massachusetts General Hospital, Boston, MA; Spaulding Rehabilitation Hospital, Mass General Brigham, Harvard Medical School, Boston, MA

## Abstract

**Introduction:**

Digital Phenotyping is the moment-by-moment quantification of the human phenotype in situ using personal digital devices. Patients who have experienced traumatic physical events often face long and complex recovery times. In non-trauma populations, digital phenotyping has already demonstrated utility in enhancing clinical outcomes, highlighting its positive potential. No prior review has comprehensively mapped the application of digital phenotyping in trauma populations. This review addresses this gap by synthesizing current evidence and exploring its role in the future of trauma rehabilitation.

**Methods:**

A systematic search of six databases (2015-2025) was conducted to identify peer-reviewed articles investigating digital phenotyping in adult (≥18 years) trauma populations. Only articles that examined personal digital devices for collecting passive data were included. Articles that solely used self-reporting scales and invasive digital phenotyping devices were excluded. Data extraction includes: (1) study population, (2) digital phenotyping modality, (3) digital endpoints, and (4) feasibility indicators. The review follows PRISMA guidelines, with risk of bias assessed with the Mixed-Methods Appraisal Tool.

**Results:**

A total of 4248 abstracts have been screened, 664 articles met inclusion criteria and are undergoing full-text review. Burn, spinal cord injury, and traumatic brain injury are the most frequently represented populations, underscoring both the relevance and need for digital phenotyping research in these populations. The most common phenotyping modality was wearable devices (accelerometers, actigraphy, and smartwatches), along with smartphone-based sensors. Digital endpoints included physical activity (duration, limb function, gait stability), sleep patterns (duration, efficiency, breathing disturbances), and physiologic parameters (resting heart rate, heart rate variability, blood pressure regulation). Feasibility outcomes were frequently reported, with most studies citing successful implementation of wearable monitoring.

**Conclusions:**

Preliminary findings indicate that digital phenotyping in trauma populations is an emerging yet underexplored field, with the potential to enhance patient care by informing frameworks for its responsible and efficient integration into trauma rehabilitation.

**Applicability of Research to Practice:**

Digital phenotyping has the power to inform the treatment of burn and trauma patient recovery by enhancing remote monitoring after injury. These tools can improve longitudinal follow-up, guide personalized treatment, and strengthen rehabilitation outcomes.

**Funding for the study:**

This work is supported by the National Institute on Disability, Independent Living, and Rehabilitation Research (#90DPBU0008).

